# Investigation of appropriate pre-analytical procedure for circulating free DNA from liquid biopsy

**DOI:** 10.18632/oncotarget.25881

**Published:** 2018-08-07

**Authors:** Akemi Sato, Chiho Nakashima, Tomonori Abe, Junichi Kato, Mitsuharu Hirai, Tomomi Nakamura, Kazutoshi Komiya, Shinya Kimura, Eisaburo Sueoka, Naoko Sueoka-Aragane

**Affiliations:** ^1^ Department of Clinical Laboratory Medicine, Faculty of Medicine, Saga University, Saga, Japan; ^2^ Division of Hematology, Respiratory Medicine and Oncology, Department of Internal Medicine, Faculty of Medicine, Saga University, Saga, Japan; ^3^ ARKRAY Inc., Kyoto, Japan

**Keywords:** liquid biopsy, pre-analytical procedure, circulating free DNA, EGFR, lung cancer

## Abstract

Liquid biopsy with circulating free DNA (cfDNA) is a recommended alternative method of re-biopsy. Quality control with cfDNA is indispensable for precise examinations, and it is desirable to achieve high-quality cfDNA separation. We investigated two issues: the influence of pre-analytical procedures on cfDNA analysis performed as a routine procedure in a standard clinical laboratory, and the extent of deterioration of cfDNA quality due to long-term storage. Comparisons among blood collection tube types, storage temperatures, and periods of blood separation were performed in terms of cfDNA quantification, cfDNA size distribution, and detection of *EGFR* mutations. Quality of cfDNA was better with collection tubes containing 3.2% sodium citrate than with those containing EDTA 2K, and was maintained with storage at 4° C for up to 72 h after blood collection, equivalent to results with cell-stabilizing blood collection tubes. Analysis of cfDNA stored for 7 years showed that samples with low allele frequency (AF) deteriorated more readily than samples with high AF. Despite the same storage period and extraction method, AF of plasma stored for 7 years was remarkably lower than that of cfDNA. However, deterioration due to long-term plasma storage was overcome by changing the DNA extraction method from a silica membrane spin column to a cellulose magnetic beads system. These results can guide the establishment of standardized pre-analytical procedures for liquid biopsy with cfDNA.

## INTRODUCTION

Detecting cancer-specific mutations by using circulating free DNA (cfDNA) is widely accepted as “liquid biopsy” for determining appropriate treatment [1]. Liquid biopsy with cfDNA is a recommended alternative method of re-biopsy, and detection of epithelial growth factor receptor (*EGFR*) mutations with cfDNA in patients with lung cancer has been approved as a companion diagnostic for EGFR tyrosine kinase inhibitors (EGFR-TKI) in Japan as well as in the US and Europe [2]. Because conventional re-biopsy requires invasive procedures, liquid biopsy with cfDNA is ideal for genotyping, especially after acquired resistance to treatment. Another advantage of cfDNA is that it reflects genomic alterations in the entire body [3]. Furthermore, recent progress in next generation sequencing (NGS) technology has led to clear demonstration of intra- or inter-tumor heterogeneity in various cancers, suggesting that tissue biopsy from a single site might not reflect genetic changes in the entire spectrum of tumors [4–10].

An important problem with liquid biopsy using cfDNA is the very small number of target mutations relative to the large amount of normal cell-derived circulating free DNA. Therefore, a highly sensitive mutation detection system is required for analysis of cfDNA [11, 12]. Several methodologies—including droplet digital PCR (ddPCR), beads-emulsion-amplification and magnetism (BEAMing), cycleave real time PCR, and NGS—have been used for the analysis of cfDNA, and these highly sensitive systems allow us to detect levels of mutations with as low as 0.1% allele frequency (AF) [13–18]. We have independently developed a fully automated, sensitive mutation detection system named mutation-biased PCR and quench probe system (MBP-QP) [18]. The detection limit of MBP-QP for the *EGFR* mutations L858R and T790M is 0.1–0.3% [19]. In a prospective multicenter observational study, T790M was observed in 40% of non-small cell lung cancer (NSCLC) patients who acquired resistance to 1^st^ generation EGFR-TKI [20].

To maximize the utility of these systems, it is essential to isolate cfDNA with a high degree of quality. In liquid biopsy, inappropriate handling of blood samples leads to difficulty in detecting low-frequency AF mutations because of contamination by genomic DNA from normal cells. Indeed, nearly 60–70% of pre-analytical errors arise from mishandling during collection and the treatment and storage of specimens [21]. Thus, pre-analytical procedures, including type of blood collection tube, choice of anticoagulant, centrifugation protocol, storage conditions, and DNA extraction method, should be reviewed. We recently reported that DNA extraction with cellulose magnetic beads produced higher recovery of cfDNA and better quality than with a silica membrane spin column system, leading to an improved efficiency of *EGF*R mutation detection [22]. However, assessment of other steps of pre-analytical procedures has not been made.

In this study we examined the influence of pre-analytical conditions on cfDNA analysis performed as a routine procedure in a standard clinical laboratory, and investigated deterioration, due to long-term storage, of cfDNA quality. As a result of these investigations, we propose appropriate pre-analytical procedures for achieving a high quality of isolated cfDNA.

## RESULTS

### Influence of anticoagulants and storage conditions on quality of cfDNA

**Figure 1:**
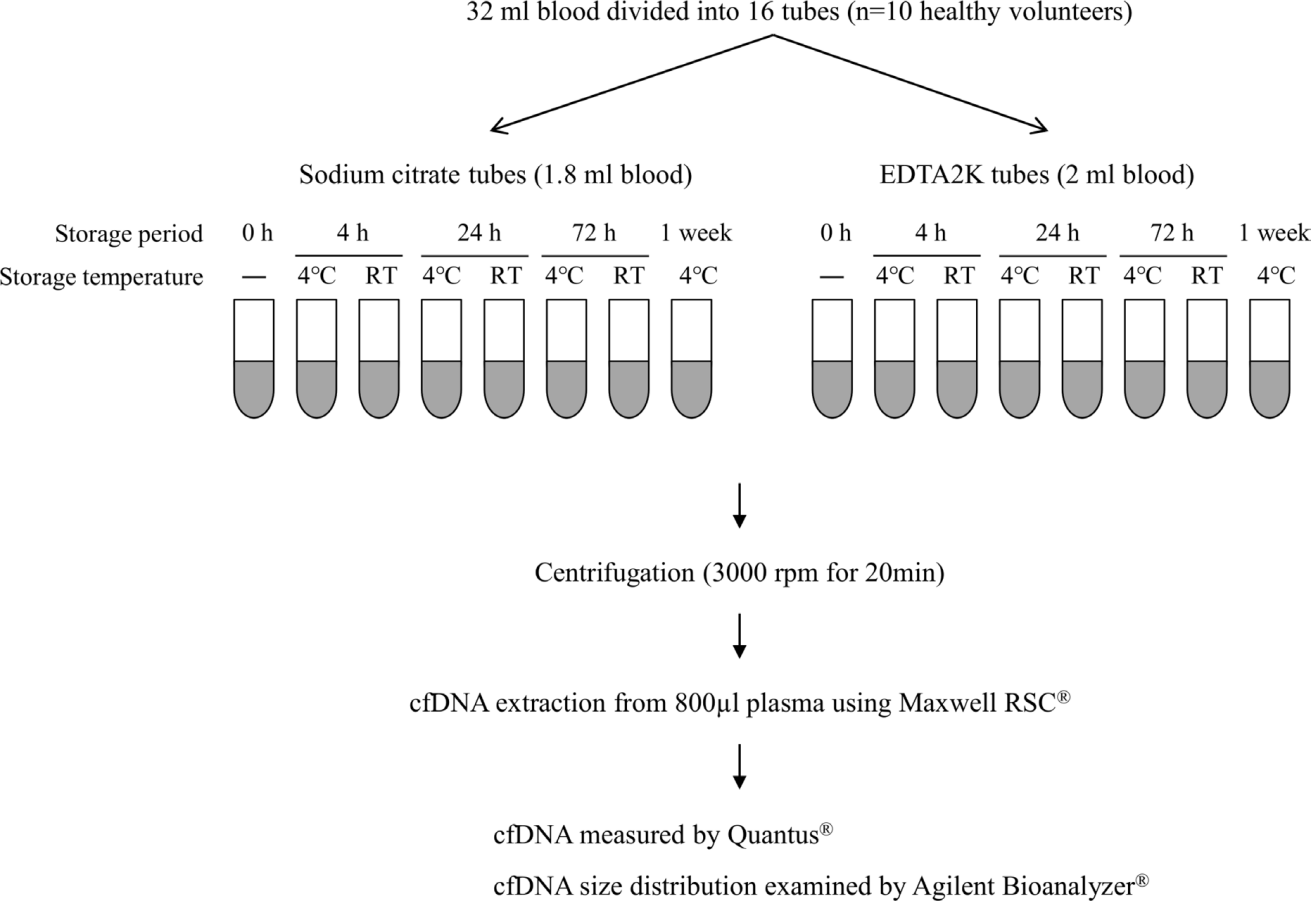
Schema for examining effects of anticoagulant and storage condition on quality of cfDNA A total of 32 ml blood was collected from each of ten healthy volunteers and divided into eight sodium citrate tubes and eight EDTA 2K tubes. Blood in each collection tube was stored at 4° C or RT for the indicated period. Plasma separation and cfDNA extraction were performed as shown in this figure. cfDNA quality was evaluated by measuring cfDNA concentration with Quantus^®^ and by analyzing cfDNA size distribution with Agilent Bioanalyzer^®^.

Thirty-two ml of peripheral blood were collected from ten healthy volunteers (three women and seven men) by standard venipuncture and divided into sixteen collection tubes: eight with sodium citrate and eight with EDTA 2K (Figure 1). Two volunteers had ages in the 40s and eight were in the 30s. Collected blood was either immediately subjected to cfDNA isolation or stored for the indicated period at 4° C or room temperature (RT) until cfDNA isolation could be performed. Plasma separation was achieved by centrifugation at 3000 rpm for 20 min at 4°C, after which 800 μL of the supernatant was subjected to cfDNA extraction.

**Figure 2:**
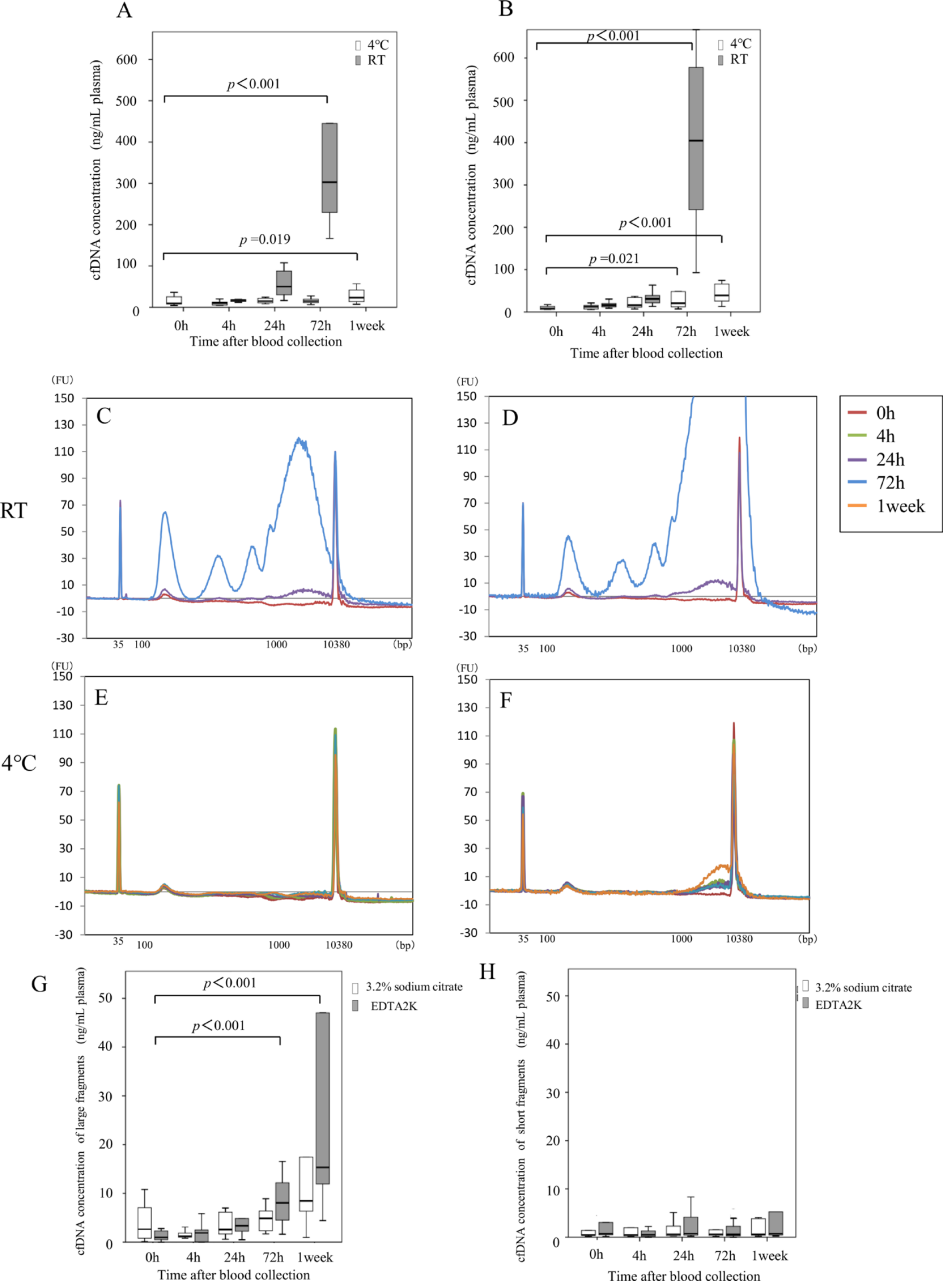
Influence of anticoagulant and blood preservation conditions on quality of cfDNA from healthy volunteers cfDNA concentrations were examined at the indicated time after blood collection using sodium citrate tubes (**A**) or EDTA 2K tubes (**B**) from ten healthy volunteers. Blood storage temperature until plasma separation was 4° C (white box) or room temperature (gray box). Size distribution of plasma DNA was analyzed with an Agilent bioanalyzer^®^; representative examples are shown in panels C-F. Sodium citrate tubes (**C**, **E**) or EDTA 2K tubes (**D**, **F**) were used for blood collection, and blood storage until plasma separation was at RT (C, D) or 4° C (E, F). DNA concentration of 1000 bp to 9000 bp fragments (**G**) and of 100 bp to 250 bp fragments (**H**) in all samples stored at 4° C was measured with an Agilent bioanalyzer^®^ as described in “Materials and methods”. Blood was collected into sodium citrate tubes (white box) or EDTA 2K tubes (gray box). Statistical analyses were performed with Friedman’s rank test.

First, we describe the effect of storage on cfDNA concentration. Results with sodium citrate tubes are shown in Figure 2A. Median cfDNA concentration immediately after blood collection was 9.3 ng/mL plasma. With blood samples kept at 4° C, median cfDNA concentration after 4, 24, and 72 h, and after 1 week, was 10.0, 15.0, 15.1, and 23.3 ng/mL plasma, respectively. With storage at RT, median concentration after 4, 24, and 72 h was 17.6, 50.1, and 302.9 ng/mL plasma, respectively. The cfDNA concentration did not change significantly until 72 h after blood collection at 4° C, whereas it was significantly elevated by 72 h at RT (*p* < 0.001). Storage for one week after blood collection resulted in significant elevation of cfDNA concentration even with storage at 4°C. With EDTA 2K tubes (Figure 2B), median cfDNA concentration immediately after blood collection was 8.8 ng/mL plasma (Figure 2B). With blood samples kept at 4° C, median cfDNA concentration after 4, 24, and 72 h, and after 1 week, was 12.4, 16.1, 20.7, and 39.0 ng/mL plasma, respectively. With storage at RT, median concentration after 4, 24, and 72 h was 16.1, 31.1, and 404.8 ng/mL plasma, respectively. cfDNA concentrations were significantly elevated at 72 h and at 1 week with storage at 4° C (*p* = 0.021) and at RT (*p* < 0.001).

Next, we describe the cfDNA size distribution. A representative result from each condition is shown in Figure 2 for sodium citrate tubes (Figure 2C, 2E) and for EDTA 2K tubes (Figure 2D, 2F). When blood was stored at 4° C, a single peak at 170 bp was observed with sodium citrate tubes (Figure 2E). However, large cfDNA from 1–9 kB appeared with EDTA 2K tubes 72 h after blood collection (Figure 2F). With storage at RT, the amount of large cfDNA increased 1 week after blood collection with both tube types (Figure 2C, 2D). Results with samples obtained from other healthy volunteers were similar (data not shown). To further evaluate cfDNA size distribution, because large fragment size is considered to represent contamination by genomic DNA, we measured cfDNA concentrations in two classes of fragment size after storage at 4° C: large fragments (1–9 kb, Figure 2G) and small fragments (100–250 bp, Figure 2H). The concentrations of large fragments after storage at 4° C were significantly higher at 72 h with EDTA 2K tubes but not with sodium citrate tubes (Figure 2G). However, concentrations of short fragments after storage at 4° C did not change until 1 week with either type of tube (Figure 2H). These results suggest that blood samples should be stored at 4° C, and plasma separation should be made within 72 h with sodium citrate tubes to maintain quality of cfDNA.

**Figure 3:**
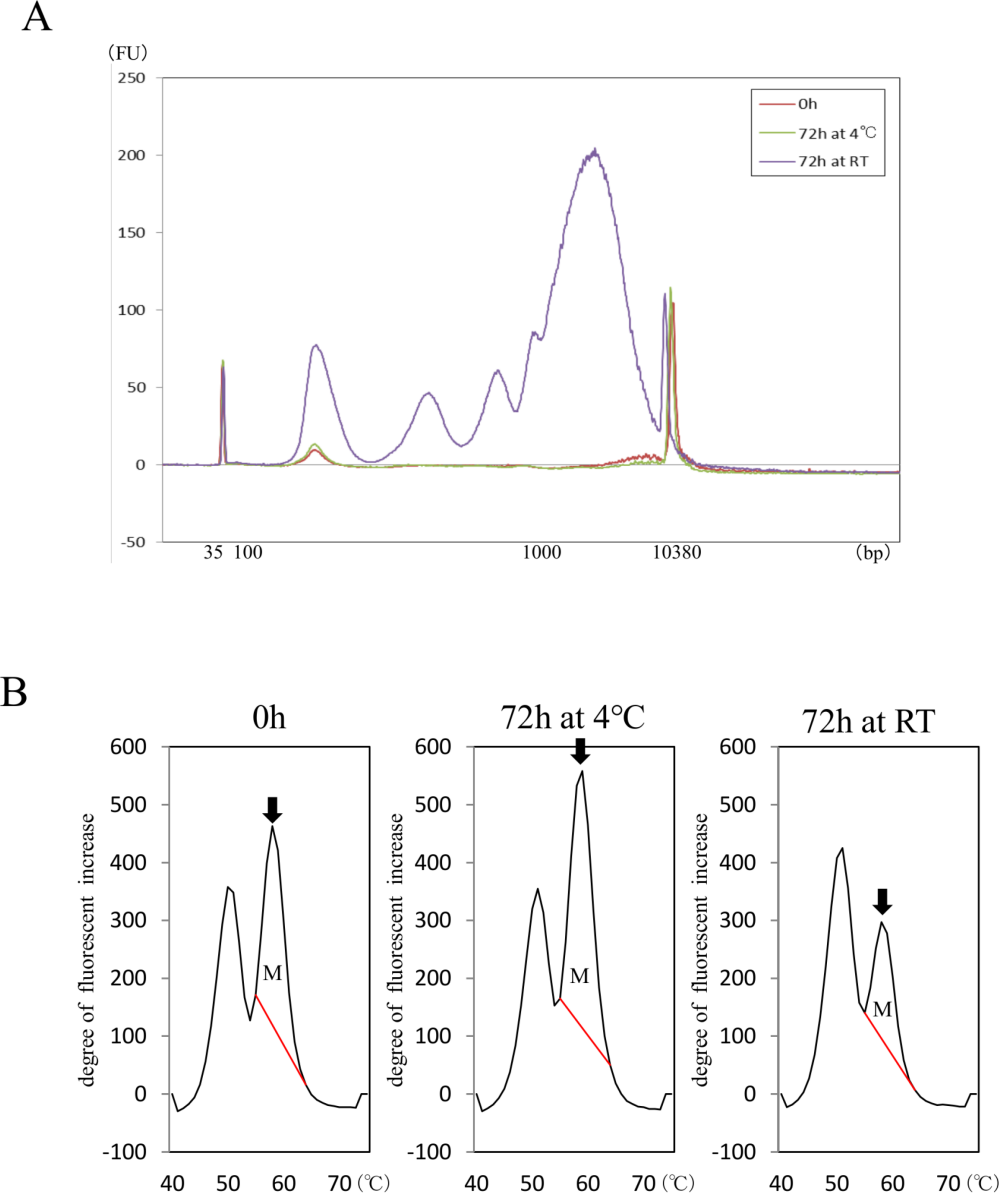
Influence of anticoagulant and blood preservation on quality of cfDNA from a patient with NSCLC harboring *EGFR* mutation cfDNA size distributions (**A**) in samples stored at 4° C or RT were compared in blood collected into sodium citrate tubes. Area under mutation peak (AUM) of L858R was examined with MBP-QP (**B**). Arrows indicate mutation peaks. AUM for L858R (indicated as M) was defined as the integral under the curve between 56° C and 68° C and was calculated by the i-densy AreaAna^®^ software.

Based on the above results, we compared effects of storage at 4° C and RT on detection of *EGFR* mutation L858R in cfDNA isolated from a patient with NSCLC after blood collection into sodium citrate tubes (Figure 3). A total of 5.4 ml of blood was collected from one patient with advanced lung cancer harboring *EGFR* L858R mutation. To minimize invasiveness, blood was collected in conjunction with clinical examination necessary for treatment. The blood was divided into 3 sodium citrate tubes: one was immediately subjected to cfDNA isolation and the others were stored at RT or 4° C for 72 h prior to cfDNA isolation. The cfDNA concentration immediately after blood collection was 7.9 ng/ml plasma. After 72 h storage, concentration was 6.9 ng/ml plasma after storage at 4° C and 140.5 ng/ml plasma after storage at RT. A relatively greater abundance of large cfDNA was observed with storage at RT, as it was in the study of healthy volunteers (Figure 3A). The amount of L858R was evaluated by measuring area under mutation peak (AUM, Figure 3B) as described in “Materials and methods”. The AUM of L858R was lower 72 h after blood collection with storage at RT, but not with storage at 4° C. In spite of the higher cfDNA concentration, that AUM was lower after RT storage suggests that leaving samples at RT results in contamination by genomic DNA and therefore leads to underestimation of genomic alterations (Figure 3B).

### Sodium citrate tubes produced cfDNA quality equivalent to that with cell-stabilizing blood collection tubes until 72 h at 4° C

First, we report on the comparison between sodium citrate tubes and EDTA 2K tubes after storage at 4° C, with two-step centrifugation, the most prevalent method in experimental laboratories [27–30]. We collected 15.2 ml blood from five additional healthy volunteers (Supplementary Figure 1) and divided it into eight collection tubes: four with sodium citrate and four with EDTA 2K. The five additional volunteers comprised three women and two men, two in their 40s and three in their 30 s. Plasma separation of blood from EDTA 2K tubes with two-step centrifugation was performed as previously reported: 1600 × g for 10 min followed by 16000 × g for 10 min [30]. Total cfDNA concentration was significantly higher after 72 h storage at 4° C with EDTA 2K tubes, but not with sodium citrate tubes (Supplementary Figure 2A, 2B). Also, large cfDNA fragments were significantly more abundant with EDTA 2K tubes after 72 h storage at 4° C (Supplementary Figure 2C), but the concentration of short fragments did not change up to 72 h after blood collection (Supplementary Figure 2D). Representative results are shown for sodium citrate (Supplementary Figure 2E) and for EDTA 2K (Supplementary Figure 2F). These results are equivalent to those for EDTA 2K tubes with single-step centrifugation, as presented above (Figure 2).

**Figure 4:**
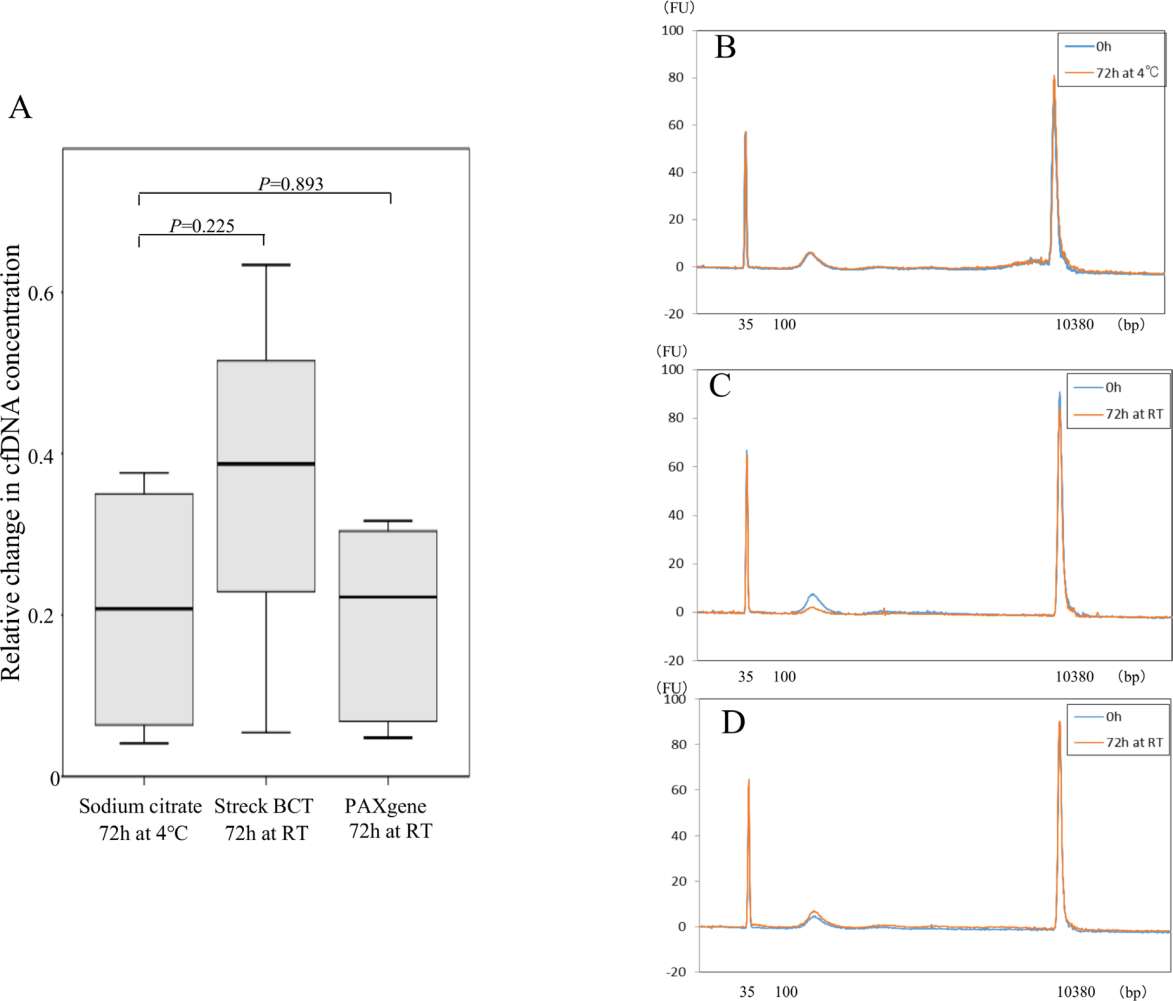
Comparison of cfDNA quality between sodium citrate tubes and cell-stabilizing tubes Relative change of cfDNA concentration in blood from five healthy volunteers was calculated as concentration after 72 h storage divided by concentration just after blood collection using sodium citrate tubes, Streck BCT, and PAXgene tubes (**A**). Statistical analyses were performed with Friedman’s rank test. Size distribution of cfDNA using the 3 kinds of blood collection tubes was analyzed with an Agilent bioanalyzer^®^; representative examples are shown for sodium citrate tubes (**B**), Streck BCT tubes (**C**), and PAXgene tubes (**D**).

Sodium citrate tubes were also compared with cell-stabilizing blood collection tubes using the blood obtained from five healthy volunteers (Supplementary Figure 3). Samples of 11.6 ml of peripheral blood were divided into six collection tubes: two each of sodium citrate tubes, Streack BCT tubes, and PAXgene tubes (Supplementary Figure 3). There were four women and one man, three in their 40 s and two in their 30 s. cfDNA was isolated 72 h after blood collection, with the sodium citrate tube kept at 4° C and the cell-stabilizing tube kept at RT, which are the recommended optimum temperatures for each respective type of tube. Plasma separation with the cell-stabilizing blood collection tubes was performed according to the manufacturer’s protocol, as shown in Supplementary Figure 3. With sodium citrate tubes stored at 4° C and cell-stabilizing blood collection tubes kept at RT for 72 h after plasma separation, relative change in cfDNA concentration (concentration after 72 h storage divided by that just after blood collection) did not differ significantly between sodium citrate tubes and Streck BCT cell-stabilizing tubes (*p* = 0.2251) or between sodium citrate tubes and PAXgene tubes (*p* = 0.893) (Figure 4A). We also did not observe any changes in size distribution of cfDNA among the three types of blood collection tubes, with the distribution containing a small single peak at 170 bp. A representative case is shown in Figure 4B–4D (B: sodium citrate; C: Streck BCT; D: PAXgene). These findings indicate that results with sodium citrate tubes are equivalent to those with cell-stabilizing tubes, in terms of cfDNA quantity and quality, for up to 72 h when the sodium citrate tubes are stored at 4° C.

### Long-term storage of cfDNA makes detection of mutation difficult

**Figure 5:**
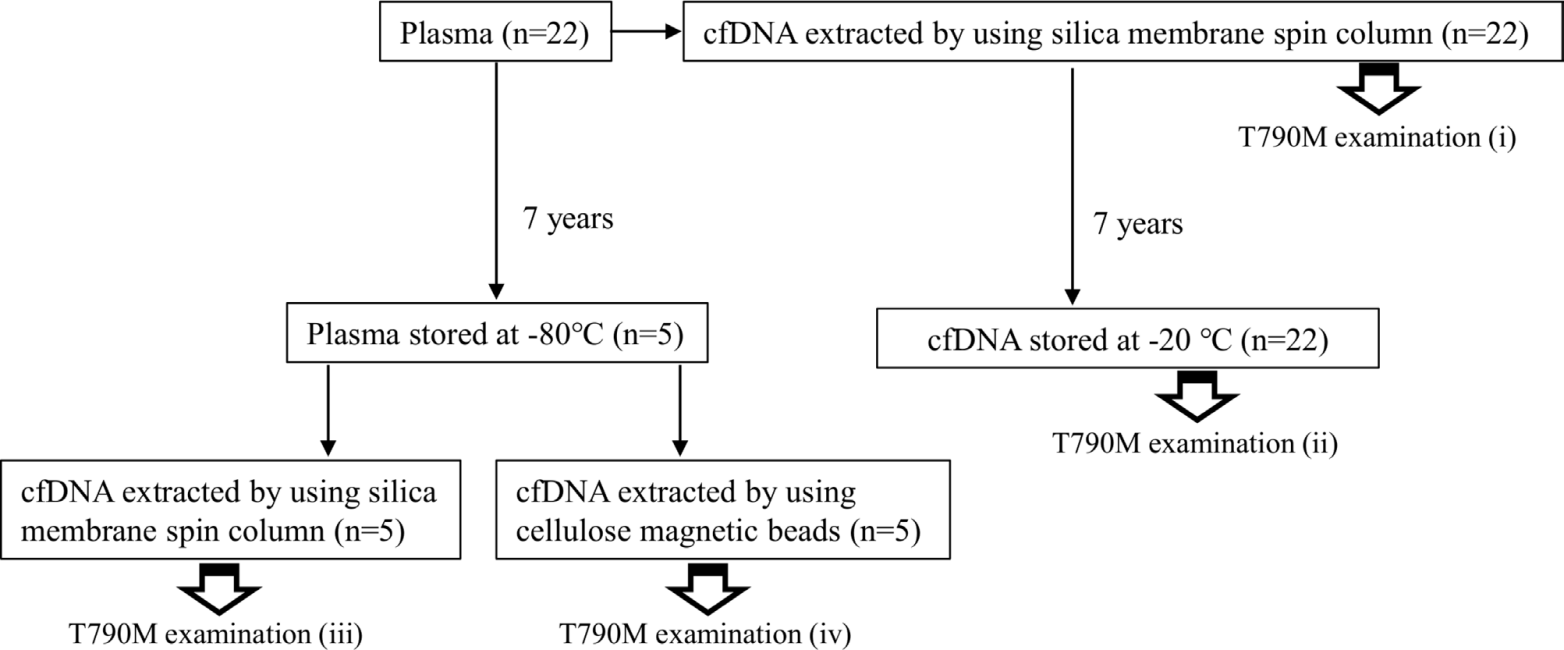
Experimental design for analysis of influence of long-term storage on cfDNA and plasma Blood samples were obtained from 22 patients with advanced lung cancer harboring *EGFR* T790M mutation detected 7 years previously. The *EGFR* T790M test was performed with MBP-QP immediately after blood collection and DNA extraction (i), and again 7 years after storage of cfDNA at −20° C (ii). Five plasma samples among the 22 patients were stored at −80° C for 7 years. cfDNA was isolated from these five plasma samples with two different isolation methods: silica membrane spin column (iii) and cellulose magnetic beads system (iv).

To investigate how long-term blood storage of cfDNA affects mutation detection, peripheral blood specimens were collected from 22 patients with advanced NSCLC treated in Saga University Hospital in 2010 (Figure 5). These patients were selected on the basis of the following inclusion criteria: 1) suffering from advanced lung cancer with distant metastasis, 2) *EGFR* T790M verified with cfDNA, and 3) cfDNA and plasma samples stored for 7 years. Plasma separation was performed immediately after blood collection and cfDNA was isolated from 200 μL plasma. Plasma was stored at −80° C (5 samples), and cfDNA was stored in low sample-to-surface-binding tubes (DNA LoBind Microcentrifuge Tubes, 1.5 mL, Eppendorf, Hamburg, Germany) at −20° C (22 samples) until further examination.

**Figure 6:**
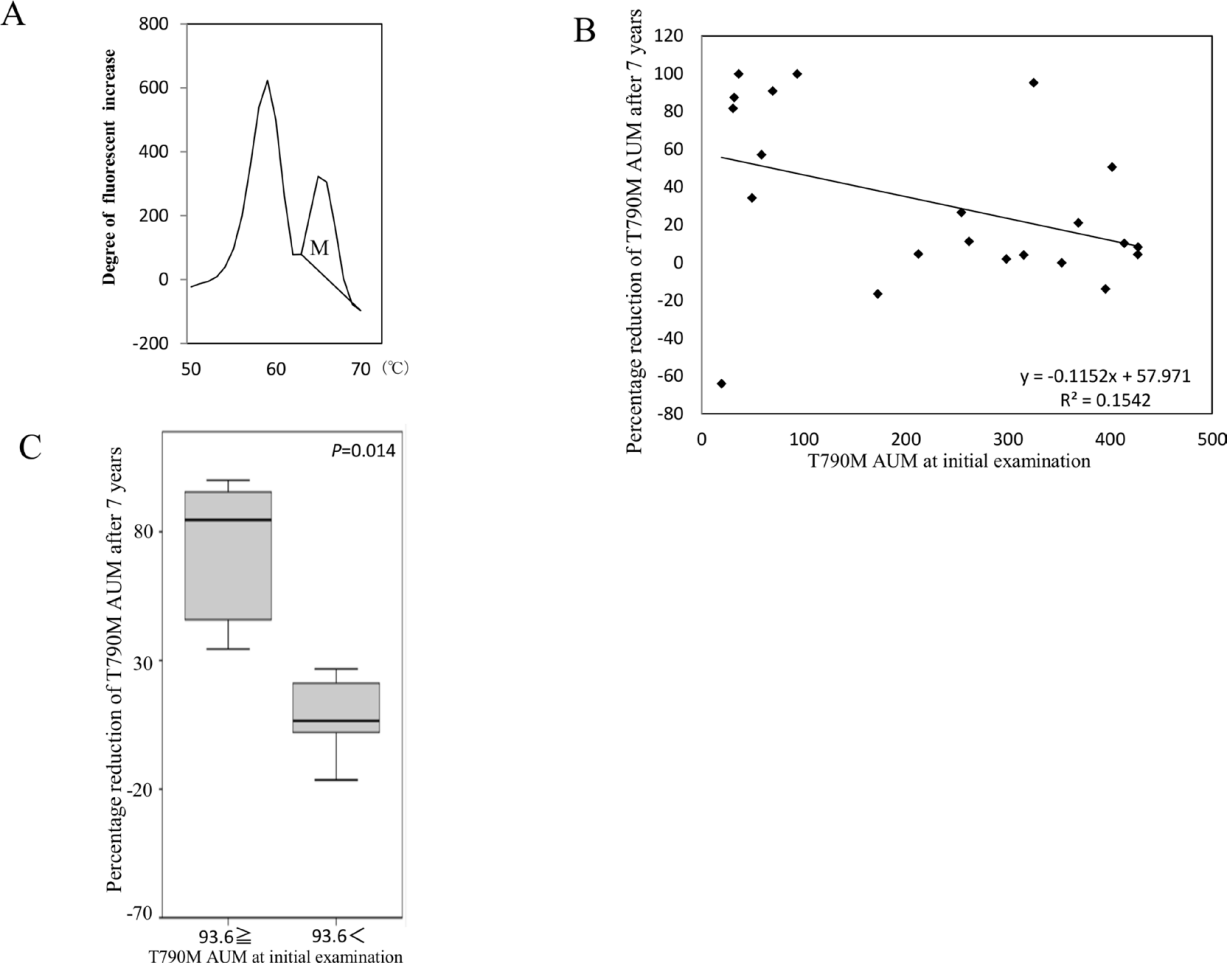
Influence of long-term storage of cfDNA on mutation detection The definition of AUM (indicated as M) is shown (**A**) The correlation between percentage reduction of T790M AUM after 7 years and the initial value of AUM (step (i) in Figure 5) was analyzed (**B**) The percentage reduction was calculated as [T790M AUM at initial examination (i) - T790M AUM after 7 years (ii)]/[T790M AUM at initial examination (i)]. Samples were divided into two groups on the basis of whether percentage reduction of T790M AUM after storage for 7 years was above or below the cut-off value 96.3 determined as the level that corresponded with 50% reduction according to the regression line (**C**) Freidman’s test was used for analysis.

First, we compared the amount of *EGFR* T790M mutation with freshly isolated cfDNA at initial examination (step (i) in Figure 5) to the amount after 7 years of storage at −20° C. The amount of T790M was evaluated by measuring AUM (Figure 6A), as described in “Materials and methods”. The percentage reduction was calculated as [T790M AUM at initial examination (i) - T790M AUM after 7 years (ii)]/[T790M AUM at initial examination (i)], and it was inversely correlated with the value of T790M AUM at initial examination (Figure 6B). According to the regression line, percentage reduction is predicted to be 50% at AUM 93.6. Percentage reduction differed significantly between the two groups defined by 93.6 as the cutpoint (*p* = 0.014; Figure 6C). These results suggest that deterioration of cfDNA occurs more easily with low AF mutations than with high AF mutations.

### Deterioration due to long-term plasma storage may be reduced by changing DNA extraction method

**Figure 7:**
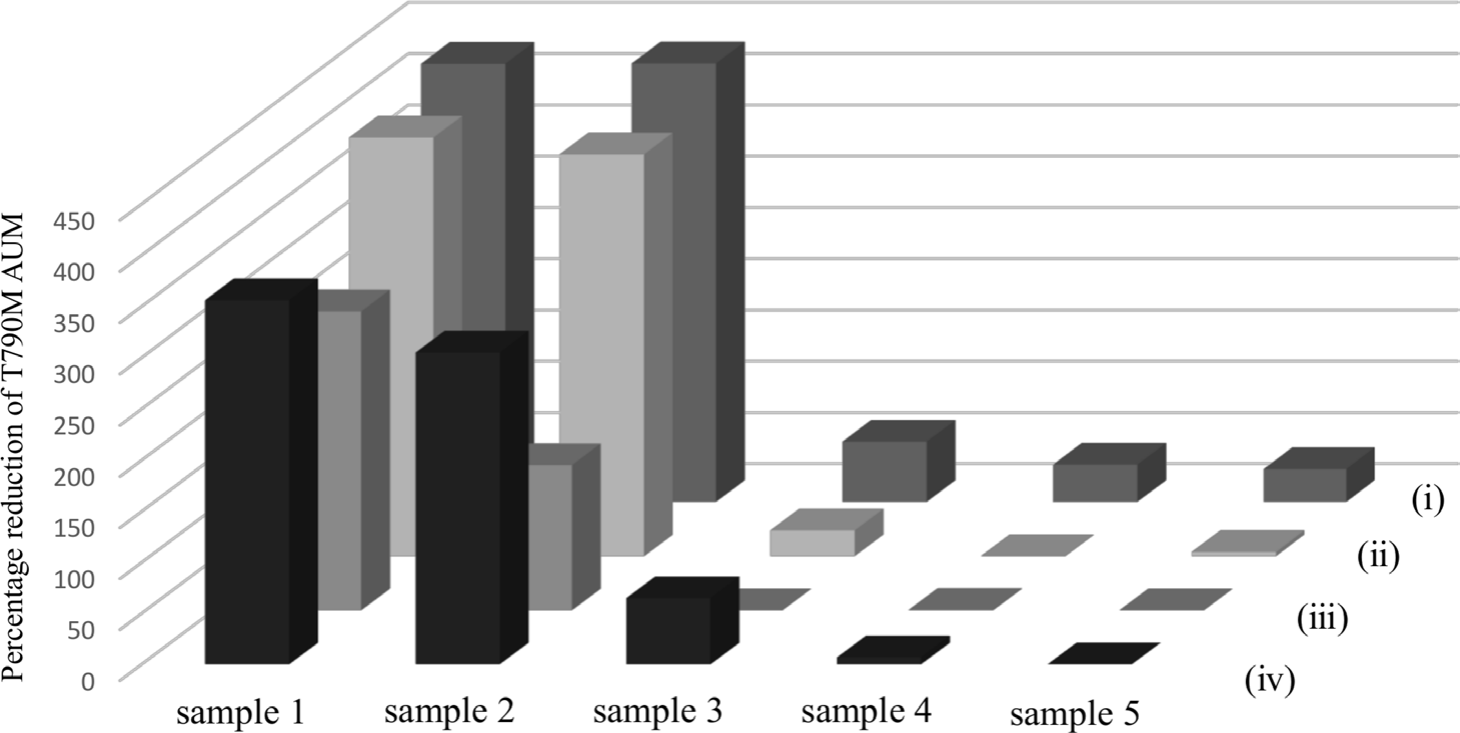
Effect of long-term plasma and cfDNA storage on cfDNA quality The workflow of this experiment is shown in Figure 5. Blood samples were obtained from five patients with advanced NSCLC carrying *EGFR* T790M mutation. The *EGFR* T790M test was performed just after blood collection (i) and 7 years after storage as cfDNA (ii). cfDNA immediately isolated from plasma was stored at −80° C for 7 years with two different isolation methods: silica membrane spin column (iii) and cellulose magnetic beads system (iv). The values of T790M AUM of these four conditions (i), (ii), (iii), and (iv) are shown for each of the five samples.

To investigate the effects of long-term plasma storage on cfDNA quality, T790M AUM with cfDNA isolated after storage from five plasma samples stored for 7 years was compared to that with cfDNA stored for 7 years (step (ii) in Figure 5). T790M AUM determined at initial examination was used as the control (step (i) in Figure 5). cfDNA from plasma stored for 7 years was isolated by two different methods: a QIAamp DNA Mini Kit^®^ with a silica membrane spin column (step (iii) in Figure 5) or a Maxwell RSC cfDNA plasma cartridge^®^ with a cellulose magnetic beads system (step (iv) in Figure 5). With all five plasma samples, the T790M peaks were highest in the initial examination (Figure 7, i) and lower with cfDNA stored for 7 years (Figure 7, ii). In spite of the same storage period and extraction method, the T790M AUM in plasma stored for 7 years (Figure 7, iii) was remarkably lower than that in stored cfDNA (Figure 7, ii). However, with the cellulose magnetic beads system (Figure 7, iv), T790M AUM in stored plasma was mostly restored even after storage for 7 years.

## DISCUSSION

High quality cfDNA for liquid biopsy is required 1) to avoid contamination by genomic DNA from white blood cells and 2) to maintain sufficient fragment length to allow the conduct of PCR-based methods. To satisfy these requirements, we assessed cfDNA quantification, cfDNA size distribution, and *EGFR* mutation detection after various pre-analytical procedures. We have shown that, to ensure quality of cfDNA, blood samples should be collected in sodium citrate tubes, storage should be at 4° C, and plasma separation should proceed no later than 72 h after blood collection. Deterioration of cfDNA with long-term storage occurs more easily with low AF than high AF mutations, but it is possible to restore sample quality with DNA extraction using the cellulose magnetic beads system.

As the first step of pre-analytical sample preparation, blood is usually collected into tubes containing EDTA, heparin, or citric acid [23, 24]. Heparin inhibits polymerase chain reaction (PCR) [25]. EDTA and citric acid maintain cfDNA stability because they inhibit DNase activity, so these anticoagulants have been used most frequently with samples intended for genetic testing [26]. Although the use of EDTA 2K tubes with 2-step centrifugation has been considered the standard method [27–30], cfDNA concentration was elevated and size distribution was shifted towards a relatively greater abundance of large fragments after 72 h storage at 4° C with that method, whereas they did not change with sodium citrate tubes. According to the results on cfDNA size distribution, large cfDNA with 1000–9000 bp was released into plasma stored at RT, which is speculated to be derived from a burst of leukocytes, leading to a relative reduction in tumor-derived cfDNA and an underestimate of mutant allele fraction. Recently, cell-stabilizing blood collection tubes—such as Streck BCT tubes and PAXgene tubes—have become commercially available [31, 32]. These collection tubes prevent cell lysis and stabilize ctDNA at RT [33]. However, our data indicate that sodium citrate tubes, which are routinely used in the clinical laboratory, produce equivalent results in terms of cfDNA quantity and quality up to 72 h after blood collection if storage is at 4° C.

Considering the spread of bio-banking, it is necessary to analyze and compare the effects, on sample quality, of long-term storage of cfDNA or plasma. In our results, *EGFR* mutation AUM in isolated cfDNA or in plasma showed 20–25% or 35–40% reduction, respectively, after 7 years of storage. This indicates that stored cfDNA is more stable than DNA in stored plasma. In addition, cfDNA isolation using a cellulose magnetic beads system is more suitable than that using a silica membrane spin column. Chan, K. C. *et al.* reported that repeated freeze-thaw cycles lead to fragmentation of cfDNA even if the DNA concentration does not change [34]. We recently demonstrated that cfDNA integrity is maintained after isolation using a silica membrane spin column [22]. This suggests that fragmentation of DNA occurs during long-term storage, leading to difficulty conducting PCR.

One of the main limitations of this study is the small sample size for examination of long-term storage. In addition, the indicator of cfDNA quality in our study depended on cancer-specific DNA mutations, such as *EGFR* T790M and L858R, which are not applicable to all patients. The need for cfDNA for comprehensive gene analysis, such as with NGS, has increased recently; therefore, precise pre-analytical procedures are required to avoid genomic DNA contamination from normal cells. In addition, a system for checking quality and deterioration of cfDNA specimens is needed, again considering the wide spread of bio-banking systems. Establishment of standardized pre-analytical procedures is therefore urgently needed.

## MATERIALS AND METHODS

### Blood collection tubes

We used tubes containing 3.2% sodium citrate (TERUMO CORPRATION, Tokyo, Japan, product number VP-CA052K), called “sodium citrate tubes” in this paper, and tubes containing EDTA 2K (TERUMO CORPRATION, Tokyo, Japan, product number VP-DK052K05), called “EDTA 2K tubes” in this paper. We also used two types of cell-stabilizing tubes: the cell-Free DNA BCT^®^ tube (Streck, Omaha, NE, USA), called “Streck BCT tubes” in this paper, and the PAXgene^®^ Blood ccfDNA tube (PreAnalytiX, Hombrechtikon, Switzerland), called “PAXgene tubes” in this paper.

### Extraction, quantification, and size distribution analysis of cfDNA

cfDNA was isolated from plasma with the Maxwell RSC ccfDNA plasma cartridge^®^ (Promega, Mannheim, Germany, product number AS 1480) or QIAamp DNA Mini Kit^®^ (QIAGEN, Valencia, CA, USA), according to the manufacturers› instructions, and stored at −80° C until further examination. Total cfDNA was quantified with the Quantus^®^ Fluorometer with a QuantiFluor^®^ dsDNA system (Promega, Mannheim, Germany). All measurements were performed three times. The size distribution of cfDNA was examined with a capillary electrophoresis system. We used the High Sensitivity DNA Kit^®^ (Agilent Technologies Inc., Santa Clara, CA, USA, Product no. 5067–4626), a microchip, and analyzed the result with an Agilent 2100 Bioanalyzer^®^ equipped with Expert 2100 software (Agilent Technologies Inc., Santa Clara, CA, USA) according to the manufacturer’s instructions. Concentrations of DNA fragments of lengths between 1000–9000 bp were normalized by bottom and top markers. The patient and healthy volunteers provided written informed consent to blood sampling and further examination. The study was conducted according to the Declaration of Helsinki. The Clinical Research Ethics Committee of Saga University Hospital approved the research protocol.

### *EGFR* mutation detection with cfDNA

The *EGFR* L858R and T790M mutations were detected with the MBP-QP method using *i*-densy™ IS 5320 (ARKRAY Inc., Kyoto, Japan), as previously reported [18, 19]. Area under mutation peak (AUM), which is correlated with allele frequency (AF), was calculated by the “idensy AreaAna” software developed by ARKRAY Inc.

### Statistical analysis

Statistical analysis was carried out with SPSS version 19 (IBM SPSS Statistics, IBM, Tokyo, Japan). To compare cfDNA concentrations in healthy volunteers, Friedman’s rank test was used. *P* < 0.05 was considered statistically significant. To evaluate long-term storage of cfDNA, correlation analysis was used to compare percentage reduction of T790M AUM with 7 years storage to that determined 7 years previously, using 50% reduction of T790M AUM to derive a cut-off value. A comparison between these two groups based on percentage reduction of T790M AUM after 7 years storage was performed with the nonparametric Mann–Whitney *U* test.

## SUPPLEMENTARY MATERIALS FIGURES


