# Supplementary material for: Investigation of appropriate pre-analytical procedure for circulating free DNA from liquid biopsy
Source: Oncotarget. 2018 Aug 7;9(61):31904–14. doi: 10.18632/oncotarget.25881 (PMC6112748; doi:10.18632/oncotarget.25881)
Supplement: Supplementary file 1 [file oncotarget-09-31904-s001.pdf]

Investigation of appropriate pre-analytical procedure for circulating free DNA from liquid biopsy

SUPPLEMENTARY MATERIALS

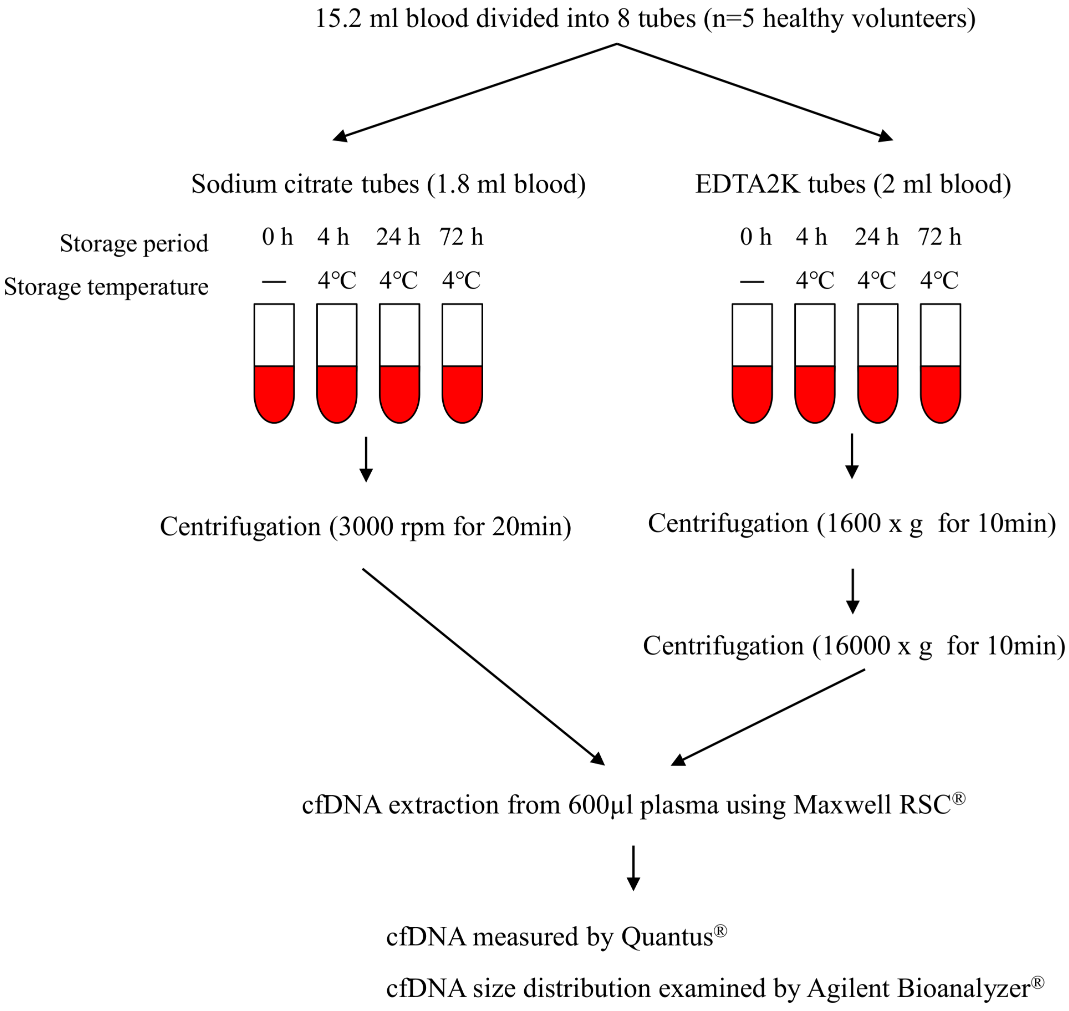

Supplementary Figure 1: The workflow of the experiment comparing sodium citrate tubes and EDTA2K tubes with two-step centrifugation. 15.2 ml blood was collected from five healthy volunteers and divided into eight tubes: four sodium citrate tubes and four EDTA 2K tubes. Plasma separation and cfDNA extraction were performed as shown in this figure. cfDNA quality was evaluated by measuring cfDNA concentration with Quantus® and by analyzing cfDNA size distribution with Agilent Bioanalyzer®.

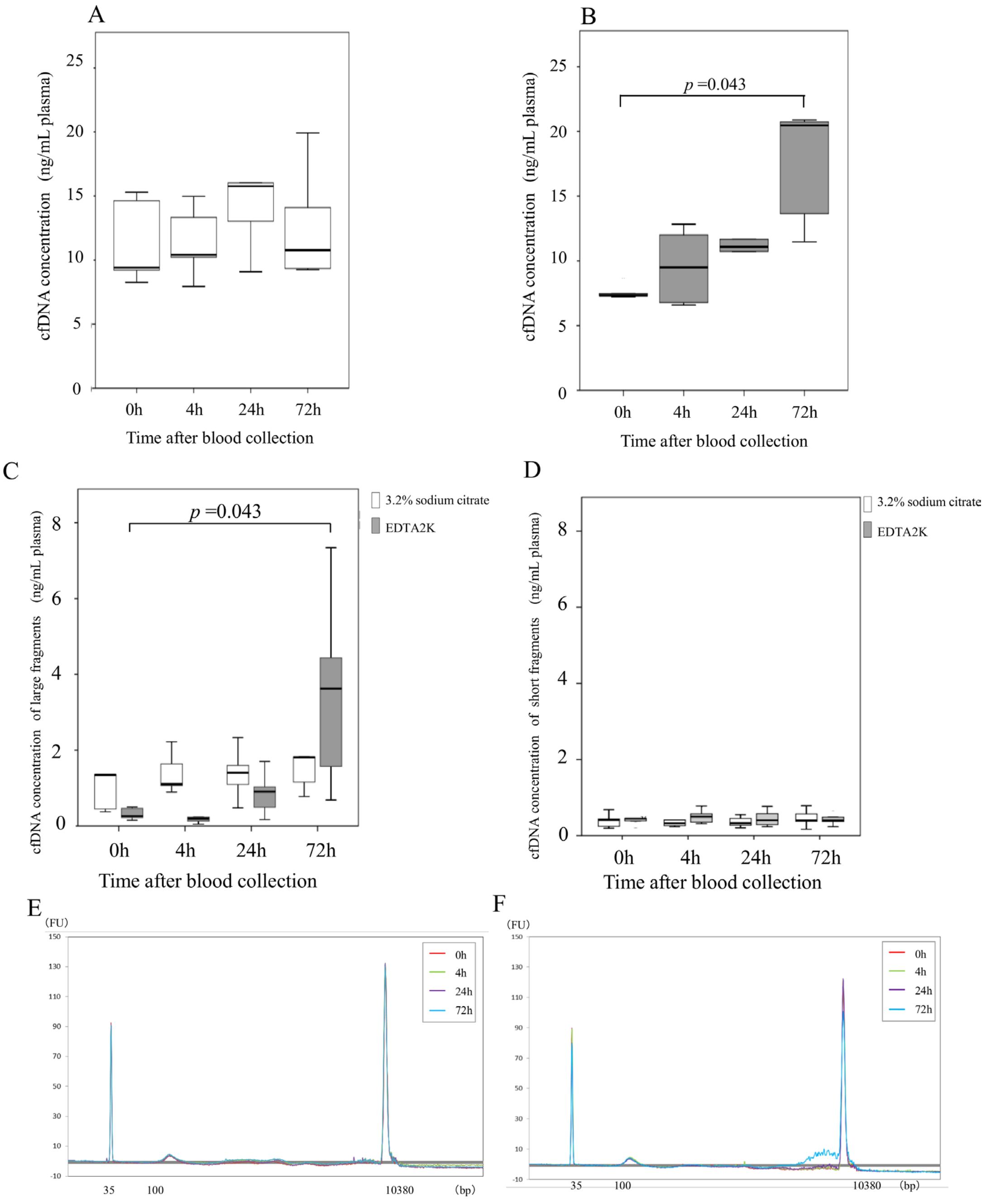

Supplementary Figure 2: Comparison of quality of cfDNA obtained from sodium citrate tubes and EDTA2K tubes with two-step centrifugation. cfDNA concentrations were examined for the indicated period of storage after blood from five healthy volunteers was collected into sodium citrate tubes (**A**) or EDTA 2K tubes (**B**). Blood was stored at 4° C until plasma separation. DNA concentration of 1000 bp to 9000 bp fragments (**C**) and of 100 bp to 250 bp fragments (**D**) in all samples stored at 4° C was measured with an Agilent bioanalyzer® as described in "Materials and methods". Representative examples are shown in panels C (sodium citrate tube) and D (EDTA 2K tube).

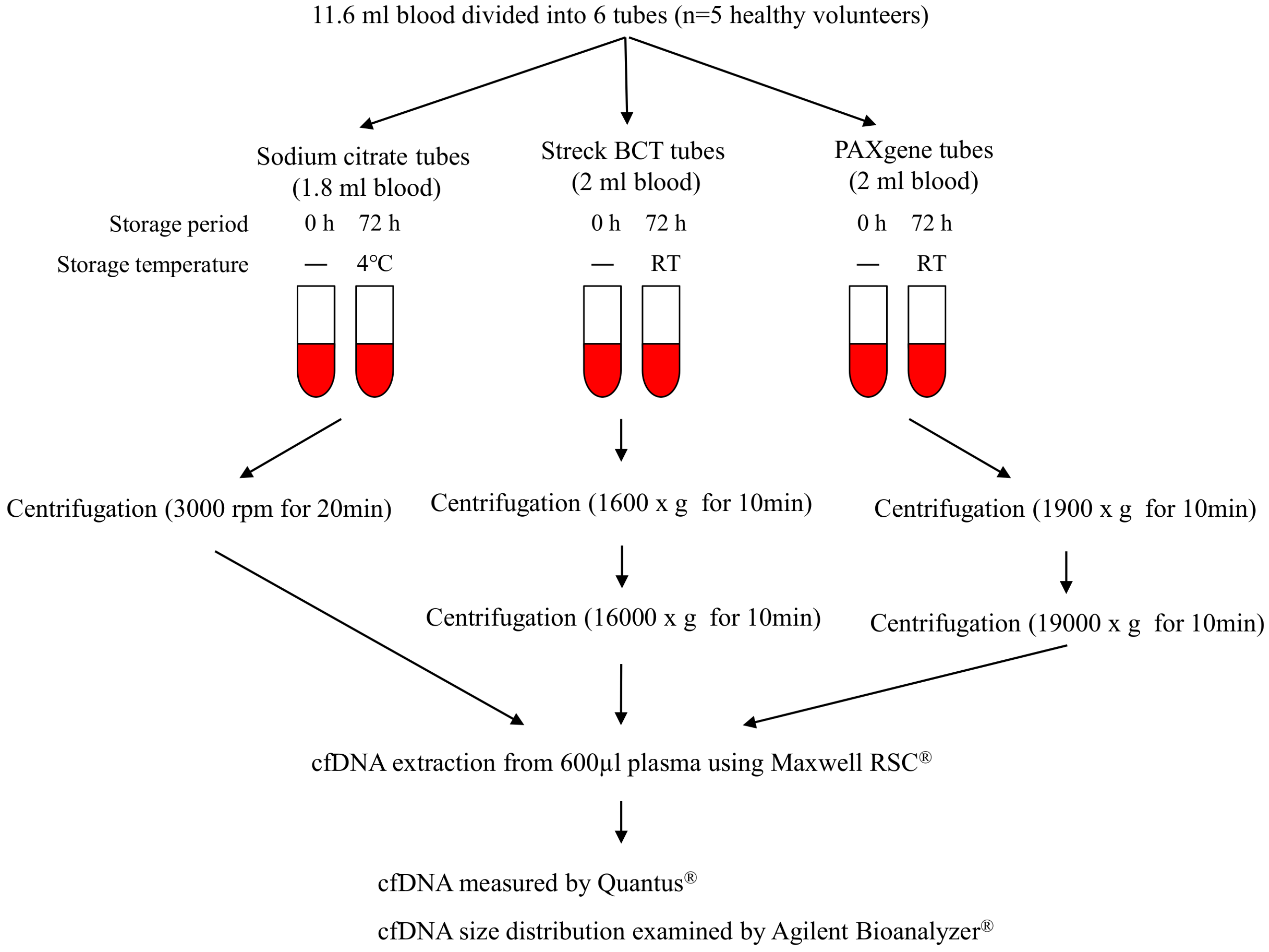

Supplementary Figure 3: Workflow of experiment to compare sodium citrate tubes and cell-stabilizing blood collection tubes. 11.6 ml of blood was collected from each of five healthy volunteers. We used sodium citrate tubes and cell-stabilizing blood collection tubes (Streck BCT tubes and PAXgene tubes). Plasma separation and cfDNA extraction were performed as shown in this figure. cfDNA quality was evaluated by measuring cfDNA concentration with Quantus® and by analyzing cfDNA size distribution with Agilent Bioanalyzer®.
